# Carbohydrate Mouth Rinsing Enhances High Intensity Time Trial Performance Following Prolonged Cycling

**DOI:** 10.3390/nu8090576

**Published:** 2016-09-20

**Authors:** Nicholas D. Luden, Michael J. Saunders, Andrew C. D’Lugos, Mark W. Pataky, Daniel A. Baur, Caitlin B. Vining, Adam B. Schroer

**Affiliations:** Human Performance Lab, Department of Kinesiology, James Madison University, Harrisonburg, VA 22807, USA; saundemj@jmu.edu (M.J.S.); dlugosac@jmu.edu (A.C.D.); patakymw@jmu.edu (M.W.P.); dab13b@my.fsu.edu (D.A.B.); viningcb@gmail.com (C.B.V.); abschroer@mix.wvu.edu (A.B.S.)

**Keywords:** cycling, endurance performance, maltodextrin, mouth wash, oralpharyngeal receptor, whey protein

## Abstract

There is good evidence that mouth rinsing with carbohydrate (CHO) solutions can enhance endurance performance (≥30 min). The impact of a CHO mouth rinse on sprint performance has been less consistent, suggesting that CHO may confer benefits in conditions of ‘metabolic strain’. To test this hypothesis, the current study examined the impact of late-exercise mouth rinsing on sprint performance. Secondly, we investigated the effects of a protein mouth rinse (PRO) on performance. Eight trained male cyclists participated in three trials consisting of 120 min of constant-load cycling (55% W_max_) followed by a 30 km computer-simulated time trial, during which only water was provided. Following 15 min of muscle function assessment, 10 min of constant-load cycling (3 min at 35% W_max_, 7 min at 55% W_max_) was performed. This was immediately followed by a 2 km time trial. Subjects rinsed with 25 mL of CHO, PRO, or placebo (PLA) at min 5:00 and 14:30 of the 15 min muscle function phase, and min 8:00 of the 10-min constant-load cycling. Magnitude-based inferential statistics were used to analyze the effects of the mouth rinse on 2-km time trial performance and the following physiological parameters: Maximum Voluntary Contract (MVC), Rating of Perceived Exertion (RPE), Heart Rate (HR), and blood glucose levels. The primary finding was that CHO ‘likely’ enhanced performance vs. PLA (3.8%), whereas differences between PRO and PLA were unclear (0.4%). These data demonstrate that late-race performance is enhanced by a CHO rinse, but not PRO, under challenging metabolic conditions. More data should be acquired before this strategy is recommended for the later stages of cycling competition under more practical conditions, such as when carbohydrates are supplemented throughout the preceding minutes/hours of exercise.

## 1. Introduction

Carbohydrate (CHO) ingestion during exercise has been widely reported to enhance exercise performance, particularly during prolonged exercise when endogenous carbohydrates are limited. A large share of the CHO-induced performance gains during prolonged exercise are thought to be due to elevated carbohydrate oxidation rates late in exercise [1,2,3,4] and/or muscle/liver glycogen sparing [3,5,6]. Additionally, there is some evidence that CHO ingestion can also better shorter-duration (<1 h or intensity >75% VO_2max_) exercise performance [4,7,8,9]; this is despite the lack of a clear metabolic advantage during these shorter exercise trials. An explanation for how CHO supplementation can improve high-intensity performance was provided by Carter and colleagues whereby CHO mouth rinsing (MR) (without swallowing) enhanced a time trial performance lasting ~1 h [10]. The authors speculated that the CHO MR affected the central nervous system by activating oropharangeal receptors. This was confirmed by subsequent work demonstrating that CHO mouth rinsing activates areas of the brain associated with reward and motor control, consequently accentuating excitatory/motor output and muscular performance [11]. 

The ergogenic value of CHO rinsing has now been examined in a variety of conditions and there is good evidence that CHO MR can enhance physical performance in certain scenarios. Interestingly, most data has indicated that the performance value of CHO MR is less prominent [12,13], and in many cases non-existent, when exercise is performed in a post-prandial/carbohydrate-fed state [12,14,15,16,17]. However, CHO MR has repeatedly been shown to elicit higher power output and faster race times in events lasting between 30 and 75 min when performed in a post-absorptive/fasted state [10,11,12,18,19,20], with three exceptions [16,21,22]. Though only examined on a few occasions, CHO MR does not typically benefit high-intensity/sprint performance [20,23,24]. The only deviation from this has been when repeated sprints were performed in a glycogen-depleted state [25], suggesting that high-intensity performance may be improved when CHO MR is delivered under conditions of fatigue or metabolic strain. Nothing is known about the potential for CHO MR to enhance short-duration trial performance at the end of prolonged exercise, analogous to a typical ‘final surge’ to the finish line late in the endurance competition. Therefore, we designed this project to test the hypotheses that mouth rinsing with CHO can enhance 2 km time trial performance when preceded by ~3 h of cycling. 

We were also interested in providing initial insight into the effects of a mouth rinse comprised of protein on late-exercise sprint performance. Though controversial, there is some evidence that adding protein to a carbohydrate supplement confers greater performance gains than carbohydrate alone [26,27,28,29,30], but the underlying physiology responsible for this observation has not been determined. Although we are not aware of general protein receptors in the oropharangeal cavity, whey protein possesses a bitter taste, and mouth rinsing with a bitter solution (quinine) can lead to higher power outputs than a placebo rinse [31]. It has also been speculated that oropharangeal receptors may be sensitive to caloric content, as ‘sweetness’ is not what confers performance gains [11]. Thus, it is conceivable that a protein mouth rinse can alter performance but this possibility has not yet been investigated. 

## 2. Materials and Methods 

### 2.1. Subjects

Eight male endurance-trained cyclists (24 ± 6 years; height, 176 ± 5 cm; weight, 74 ± 7 kg; VO_2max_, 63.8 ± 5.7 mL·kg^−1^·min^−1^) participated in this project. All subjects performed ≥3 days of cycling per week for ≥2 months prior to study enrollment. All participants were informed about study procedures and potential risks prior to consent. All procedures were approved by the James Madison University Institutional Review Board (IRB #14-0119). 

### 2.2. Cardiorespiratory Fitness

Subjects performed an incremental exercise test to exhaustion on a bicycle ergometer (Velotron; Racermate, Seattle, WA, USA) to determine maximum oxygen uptake (VO_2max_) and power output at VO_2max_ (W_max_). Subjects began the protocol at a self-selected power output that would be “a manageable workload during a 60 min ride”. Power was then increased by 25 watts (W) every 2 min until volitional exhaustion. Breath samples were continuously monitored with a calibrated Moxus Modular Metabolic System (AEI Technologies, Pittsburgh, PA, USA) and data were aggregated in 30 s increments. Peak power (W_max_) in the final completed stage was used to prescribe exercise intensity for subsequent exercise trials. 

### 2.3. Experimental Design

**Figure 1:**
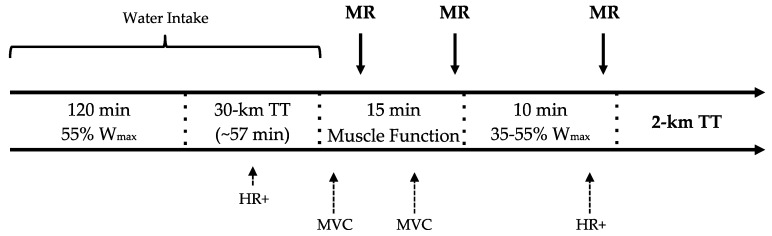
Experimental trial design with dependent measures. Water intake bracket indicates when only water was provided; HR+ with short arrow indicates heart rate (HR), rating of perceived exertion (RPE), and glucose measurements taken midway through the 30 km time trial (TT) prior to the mouth rinse (MR) intervention; HR+ with tall arrow indicates HR, RPE, and glucose measurements taken to determine the effects of the MR intervention; MVC, maximum voluntary.

The study was carried out in a counterbalanced, double-blinded fashion, with trials separated by seven to 10 days each. Following cardiorespiratory fitness testing, subjects completed 1 familiarization trial and three experimental trials on the aforementioned cycle ergometer. Each trial consisted of: 120 min of constant-load cycling at 55% W_max_, a simulated 30 km time trial (TT) (~57 min), 15 min of rest that included peak isometric force testing, 10 min of constant-load cycling (3 min at 35% W_max_ and 7 min at 55% W_max_), and a computer simulated 2 km time trial (~4 min) (Figure 1). The inclusion of a variable and self-selected intensity 30 km time trial prior to the mouth rinse intervention instead of continuing constant-load cycling allowed us to assess 30 km TT reliability under placebo (water) conditions, as we have reported elsewhere [32]. This decision may have introduced additional variability for subsequent measures, though data gathered during the 30 km TT and finishing times indicate that subjects experienced similar physiological stimuli prior to each of the three MR treatments. 

Subjects were provided with 600 mL of water immediately prior to each trial, after which 150 mL of water was provided every 15 min during the 120 min steady-state ride and at three points during the 30 km TT (7.5 km, 15 km, and 22.5 km). Subjects were asked to void their bladders prior to all trials. A fan was placed ~2 m from the handlebars on high speed setting for uniform cooling during each trial and trials were conducted under thermoneutral conditions. All trials were performed in the morning, with no more than one hour separating start times for a given subject, two hours after consumption of a standardized breakfast (Section 2.6). 

### 2.4. Mouth Rinse Solutions

The MR treatments contained 100 mL of deionized water (PLA) with the addition of either 6.4 g of maltodextrin (NOW Foods, Bloomingdale, IL, USA) or 6.4 g of hydrolyzed whey protein isolate (AMCO, Burlington, NJ, USA). Commercially available stevia was used to minimize differences in taste and smell. 

During the three experimental trials (T1, T2, T3), subjects received 25 mL of each respective mouth rinse. Rinses were administered at three time points during the exercise trial: minutes 5:00 and 14:30 of the rest phase and at minute 8:00 of the 10 min constant-load segment (see Figure 1). Subjects swirled each rinse in their mouth for 5 s after which it was expectorated. 

### 2.5. Dependent Measures

#### 2.5.1. Cycling Performance

Finishing time from each 2 km cycling time trial was used as the performance measure.

#### 2.5.2. Skeletal Muscle Function

Isosometric peak torque (Maximum Voluntary Contraction, MVC) of the knee extensors was assessed at minutes 1:00 and 10:00 of the 15-min rest phase prior to the 2 km TT, as previously described [33]. Subjects performed three maximal attempts per test, each lasting 3 s, with one minute of rest between attempts. Peak force (N) was determined by the highest value from the three attempts, without visual feedback. While the 15 min rest period detracts from the ecological validity of the experimental design, it was necessary to facilitate muscle function testing before and after the MR intervention. 

#### 2.5.3. Heart Rate (HR), Rating of Perceived Exertion (RPE), and Blood Glucose

Heart rate was monitored throughout the duration of each exercise session using a chest-worn heart rate monitor (Suunto, Vantaa, Finland). Finger-stick blood samples were obtained at minute 8 of the 10 min constant-load phase. Glucose levels were determined immediately from whole blood using an automated analyzer (YSI 2300 STAT glucose, Yellow Springs, OH, USA). Subjective ratings of exertion using the Borg RPE scale were obtained simultaneous with blood sampling. Heart rate, RPE, and glucose were also assessed midway through the 30-km TT prior to the MR intervention.

### 2.6. Dietary and Physical Activity Controls

Subjects were instructed to maintain normal dietary habits throughout the study and were also provided with a standardized breakfast that was consumed 2 h prior to each trial (500 kcal; 90–100 g carbohydrate; 8–12 g protein, and 4–8 g of fat). Subjects completed a diet record for 24 h preceding the first experimental trial. Using a copy of the initial diet record, subjects were instructed to replicate their diet for the 24 h prior to each subsequent experimental trial. Subjects were instructed to refrain from heavy exercise for 48 h preceding each treatment trial, and were instructed to maintain consistent exercise habits between each of these trials. Subjects were also instructed to abstain from alcohol and caffeine for 24 and 12 h prior to each trial, respectively.

### 2.7. Statistical Analysis

All data were log transformed to diminish the effects of non-uniformity. Magnitude-based inferences about the data were derived using methods described by Hopkins and colleagues [34]. For performance, a previously established “smallest worthwhile change” in performance was used as the threshold value for a substantial treatment effect (CHO and PRO vs. PLA) [35]. The smallest worthwhile change in performance was defined as 0.3% of the within-subject variability of select groups of elite cyclists across repeated time trials (Coefficient of Variation = 1.3% for time), which translates to 0.8 s for the current project [36]. Published spreadsheets [37] were then used to determine the likelihood of the true treatment effect (of the population) reaching the substantial change threshold (0.8 s); these percent likelihoods were classified as <1% almost certainly no chance, 1%–5% = very unlikely, 5%–25% = unlikely, 25%–75% = possible, 75%–95% = likely, 95%–99% = very likely, and >99% = almost certain. Clinical inference criteria were used to classify the effects of treatment on performance. Specifically, if the percent chance of the effect reaching the substantial change threshold was <25% and the effect was clear, it was classified as “trivial.” If the percent chance of the effect reaching the substantial change threshold for benefit exceeded 25% but the chance for harm was >0.5% the effect was classified as unclear. An exception to the 0.5% chance of harm criterion was made if the benefit/harm odds ratio was >66, in which case the effect was interpreted as clear and an inference was assigned. 

For all other variables, the classification system detailed above was applied. However, the “smallest worthwhile change” was determined for each variable by multiplying the within-subject standard deviation under PLA conditions by 0.2. Further, mechanistic criteria were used such that if 90% confidence intervals included values that exceeded the substantial change threshold for both a positive and negative effect, effects were classified as unclear (>5% chance of reaching the substantial threshold for both a positive and negative effect), with no exceptions. For ease of interpretation, *p*-values derived from simple contrasts between treatments are included alongside the magnitude-based inferential outcomes.

## 3. Results

### 3.1. Cycling Pre-Load

As previously described, prior to MR intervention, subjects performed 120 min of constant-load cycling at 55% W_max_ (190 ± 22 Watts) followed by a 30 km TT. Data obtained midway (15 km) through the 30 km TT and finishing times are displayed in Table 1.

### 3.2. Mouth Rinse Effects

#### 3.2.1. Performance

**Figure 2:**
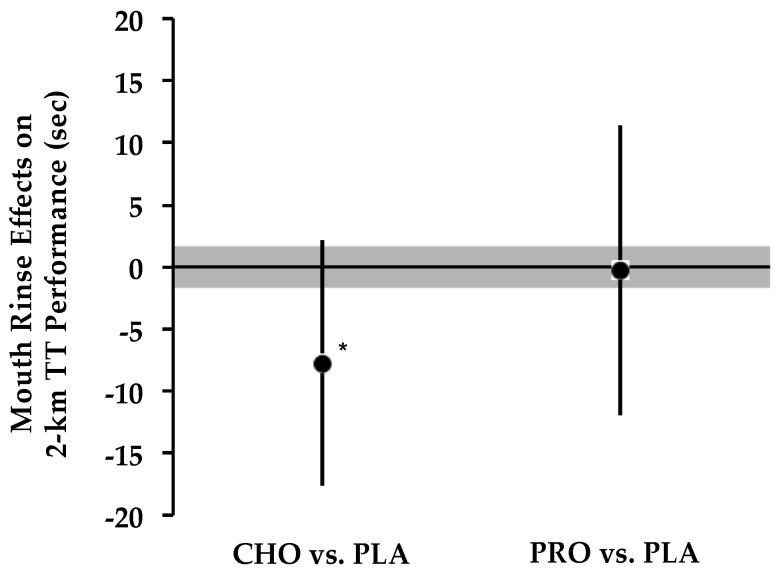
Effect of CHO and PRO mouth rinses on 2 km time trial (TT) Performance. Circles represent mean treatment difference compared to placebo. Bars depict 90% confidence interval. Shaded area notates threshold value for smallest meaningful effect. * ‘Likely’ faster than PLA.

CHO MR ‘likely’ enhanced the 2 km TT performance by 3.8% ± 4.7% (*p* = 0.11) compared to PLA, whereas the comparison between PRO vs. PLA was ‘unclear’ (0.4% ± 5.6%; *p* = 0.91). The 2 km TT data are displayed in Table 2 and Figure 2.

#### 3.2.2. Skeletal Muscle Function

The MVC ‘likely’ increased from 456 ± 108 N to 490 ± 106 N during the 15 min muscle function period with PLA (7.9% ± 5.8%; *p* = 0.03), ‘possibly’ increased from 445 ± 61 N to 470 ± 70 N with CHO (5.3% ± 3.8%; *p* = 0.03), but had only a ‘likely trivial’ increase from 466 ± 84 N to 475 ± 75 N with PRO (2.5% ± 4.2%; *p* = 0.78). There was a ‘possibly trivial’ (68% likelihood; *p* = 0.49) difference between the change in MVC with PLA compared to CHO, whereas PRO had a ‘possibly’ smaller increase in MVC compared to PLA (57% likelihood; *p* = 0.09). Changes in MVC during the 15 min period are displayed in Table 2. 

#### 3.2.3. Heart Rate, RPE, and Blood Glucose

Heart rate during the 10 min constant-load phase was ‘likely’ higher with CHO compared to PLA (3.8% ± 5.3%; *p* = 0.19). The comparison between PRO and PLA was ‘unclear’ (*p* = 0.73). Differences in RPE between MR treatments were ‘unclear’. Blood glucose levels during the 10 min constant-load phase were ‘likely’ higher with CHO relative to PLA (5.8% ± 4.1%; *p* = 0.02), and likely lower with PRO (−5.3% ± 7.0%; *p* = 0.20) compared to PLA. Mean HR, RPE, and blood glucose levels are displayed in Table 2.

## 4. Discussion

This project was primarily designed to examine the potential for a carbohydrate (CHO) mouth rinse to enhance high-intensity performance towards the end of prolonged exercise. We also included a separate experimental trial to investigate the possible benefit of mouth rinsing with a protein solution (PRO). The CHO mouth rinse ‘likely’ improved the 2 km TT performance (vs. PLA), whereas there were no systematic differences in the TT performance between PRO and PLA. This is the first evidence that late-exercise cycling TT performance can be improved by a CHO rinse, suggesting that endurance cyclists should consider CHO mouth rinsing to optimize performance during the late stages of competition, particularly when CHO supplementation is limited throughout exercise. The PRO MR data infers that previously reported benefits of adding PRO to a CHO supplement during prolonged exercise were not facilitated by detection of PRO in the mouth [26,27,28,29,30]. The ineffectiveness of the PRO rinse also indicates that physical gains from the CHO MR are specific to the presence of CHO.

The unique observation in this study is that the CHO MR enhanced short-duration TT performance lasting only ~3 min. Most previous work has demonstrated that CHO MR has little impact on short-duration, high-intensity ‘performance’ [20,23,24]. The only exception to this is the recent report that a repeated run-sprint performance was moderately enhanced by CHO MR, on the morning after a glycogen-depleting protocol [25]. Collectively, it now appears that MR can be effective for high-intensity exercise performance under difficult glycometabolic conditions. This is generally consistent with what is known about the efficacy of CHO rinsing during performances lasting longer than 30 min. The most pronounced improvements with CHO MR have been observed when exercise is commenced in a fasted state and when carbohydrate supplementation is withheld throughout exercise [12,13,38]. Further, the evidence supporting the performance advantages of CHO rinsing is much more consistent when the exercise is performed in a fasted state [10,11,12,18,19,20], in contrast to the null findings typically observed when the exercise is conducted in a fed state [12,14,15,16,17]. This indicates that the degree to which the presence of the carbohydrate in the mouth activates the reward/pleasure centers of the central nervous system (CNS) is related to carbohydrate availability or feeding latency time; specifically, that the CNS response to the presence of carbohydrate in the mouth is magnified under conditions of substantial energetic stress and/or when more time has elapsed since the most recent carbohydrate feeding. The current data would seem to support this hypothesis, though we did not directly examine CNS activity. 

As mentioned earlier, CHO MR is believed to enhance physical performance by activating areas of the brain associated with reward/pleasure, thereby increasing excitatory/motor output and muscular performance. One criterion measure of this response is peak muscle strength, and though not unanimous [39], mouth rinsing with CHO can partially restore peak strength following fatiguing contractions [40], following 60 min of cycling [41], as well as attenuate force diminution during sustained muscle contractions [42]. We evaluated peak torque during a maximum voluntary contraction before and after the MR treatment, following the prolonged cycling stimulus. In contrast to our hypothesis, the CHO MR (+25 N) did not increase knee extensor torque more so than PLA (+34 N). The discrepancy between this and previous reports may be due to treatment dosing, specifically that the mouth rinse dose that separated pre- and post-strength measurements here may not have been enough to elicit a treatment effect. Strength measurements obtained in the current project were assessed before and after a single dose swirled in the mouth for 5 s, which is much shorter than the 10 s [40] and 60 s [42] rinses provided in the studies documenting strength improvements. This explanation is strengthened by a previous report that endurance performance is enhanced to a greater extent following 10 s compared to 5 s rinses [43]. Additionally, the decline in skeletal muscle force following 60 min of cycling was attenuated with a 5 s CHO MR, but the rinse was delivered seven times throughout the exercise session [41]. Therefore, additional mouth rinses or longer swill time may have been required to elicit strength gains. The obvious disconnect in the current dataset is that despite the absence of a CHO treatment effect on MVC, the CHO rinse did improve the 2 km TT performance. However, while the strength measurements were obtained after a single mouth rinse, two subsequent doses were administered prior to the 2 km TT. 

Somewhat unexpectedly, the CHO MR was also associated with slightly elevated blood glucose levels and heart rate during constant-load cycling prior to the 2 km TT. Blood glucose following a CHO MR has been assessed during variable intensity exercise on a number of occasions and it has consistently been unaffected [12,38,44]. Though this possibly suggests that the rise in blood glucose resulted from the digestion of the MR constituents, we are inclined to believe that it was a centrally mediated response to the MR. The primary ingredient in the CHO MR, maltodextrin, cannot be digested into glucose residues in the oral cavity [45]. Further, investigators were present to administer the MR treatments and to ensure that the rinse was expectorated and not swallowed. In addition, while we did not measure the volume of the expectorate, any small amount of solution that may have been ingested was likely insufficient to significantly raise blood glucose levels. In support of a centrally mediated MR effect on blood glucose, the only other paper that measured blood glucose during constant-load exercise also noted elevated blood glucose levels [17]. The authors speculated that the increase in blood glucose following the CHO MR might have been due to an increase in hepatic glucose output facilitated by activation of hepatic and/or pancreatic sympathetic nerve activity [46]. Regardless of the mechanisms, much like the previous authors, we doubt the physiological relevance of the small differences in blood glucose levels (~3 mg/dL). There is good evidence that even larger discrepancies in blood glucose do not necessarily translate to a faster cycling performance [10,47,48]. Furthermore, the difference in blood glucose levels between PRO and PLA MR trials in the current study was also ~3 mg/dL, and the TT performances were virtually identical. Like blood glucose, heart rate was also slightly elevated during constant-load exercise following the CHO MR. There is no precedent for this response but it could also be related to an increase in sympathetic nerve activation mentioned earlier. It is also possible that the CHO MR led to an increase in cycling cadence, which has been shown to increase heart rate even when power output is clamped [49]. Unfortunately we do not have cadence data to test this hypothesis. 

## 5. Conclusions 

Here we present the first evidence that cyclists may want to consider mouth rinsing with a CHO solution prior to the closing kilometers of a competitive event. The practical relevance of this finding should be further established, as the mouth rinse solution was administered after approximately three hours of cycling without carbohydrate supplementation.

## Figures and Tables

**Table 1:** The 30 km time trial prior to mouth rinse intervention.

Condition	Heart Rate (bpm)	RPE	Blood Glucose (mg/dL)	Finishing Time (min)
**Placebo**	150 ± 14	16 ± 1	68 ± 80	57.02 ± 5.21
**Carbohydrate**	148 ± 14	16 ± 1	68 ± 70	57.04 ± 2.75
**Protein**	144 ± 15	16 ± 1	66 ± 10	57.89 ± 7.38

Values are expressed as means ± SD. Data were obtained midway (15 km) through the 30 km TT. RPE = rating of perceived exertion. Data demonstrates that similar workloads were performed prior to the mouth rinse intervention.

**Table 2:** The 2 km performance, peak strength, and heart rate, rating of perceived exertion, and glucose.

Condition	∆ MVC (N)	Constant-Load—55% W_max_			2 km TT Finishing Time (s)
		Heart Rate (bpm)	RPE	Blood Glucose (mg/dL)	
**PLA**	34 ± 40	150 ± 13	14 ± 20	59 ± 8	200.1 ± 10.8
**CHO**	25 ± 25	156 ± 10 *	14 ± 20	63 ± 7 **	192.4 ± 8.2 ^††^
**PRO**	10 ± 26 ^‡^	152 ± 60	14 ± 3	56 ± 10 ^†^	199.9 ± 18.4

Values are expressed as means ± SD. ∆ MVC, change in MVC from beginning to end of 15 min muscle function phase. RPE = rating of perceived exertion, MVC = maximum voluntary contraction. Statistics were used to separately compare PLA to CHO and PRO. Magnitude-based inferences for treatment comparisons are notated as follows: ^‡^ PRO vs. PLA, Possible (57%); * CHO vs. PLA, Likely (77%); ** CHO vs. PLA, Likely (92%); ^†^ PRO vs. PLA, Likely (77%); ^††^ CHO vs. PLA, Likely (89%).
